# Supplementary material for: Efficient Nanocrystal Photovoltaics with PTAA as Hole Transport Layer
Source: Nanomaterials (Basel). 2022 Sep 3;12(17):3067. doi: 10.3390/nano12173067 (PMC9458081; doi:10.3390/nano12173067)
Supplement: Supplementary file 1 [file nanomaterials-12-03067-s001.zip › nanomaterials-1870861-supplementary.pdf]

nanomaterials

Supplementary Materials

Efficient Nanocrystal Photovoltaics with PTAA as Hole Transport Layer

Ao Xu [1], Qichuan Huang [1], Kaiying Luo [1], Donghuan Qin [1,*], Wei Xu [1], Dan Wang [1] and Lintao Hou [2]

[1] State Key Laboratory of Luminescent Materials & Devices, Institute of Polymer Optoelectronic Materials & Devices, South China University of Technology, Guangzhou 510640, China
[2] Guangdong Provincial Key Laboratory of Optical Fiber Sensing and Communications, Guangzhou Key Laboratory of Vacuum Coating Technologies and New Energy Materials, Siyuan Laboratory, Department of Physics, Jinan University, Guangzhou 510632, China
* Correspondence: qindh@scut.edu.cn; Tel.: +86-020-8711-4346

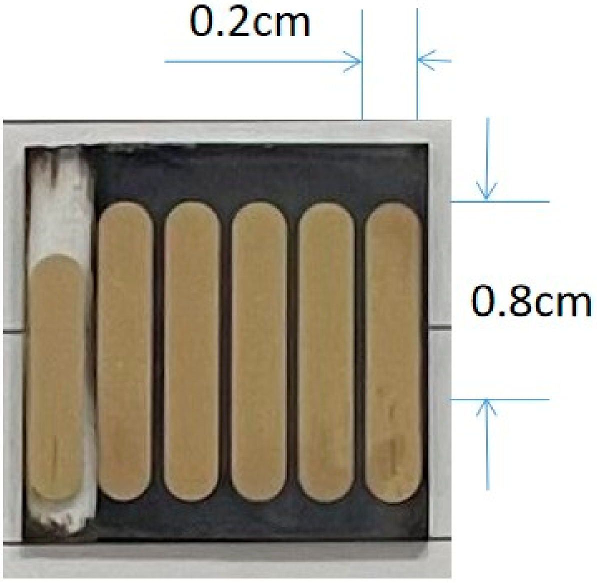

Figure S1. The photo of a real CdTe solar cell. In the left part of cathode, the ZnO/CdS/CdSe/CdTe active layer has been scraped away before Au deposition. The active area is 0.16 cm^2 for each device.

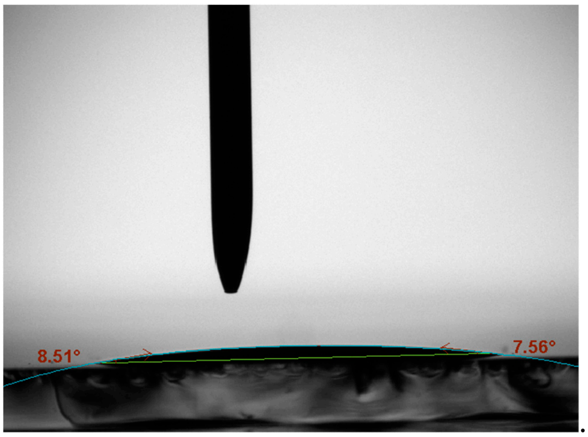

Figure S2. The contact angle of PTAA on the CdTe film.

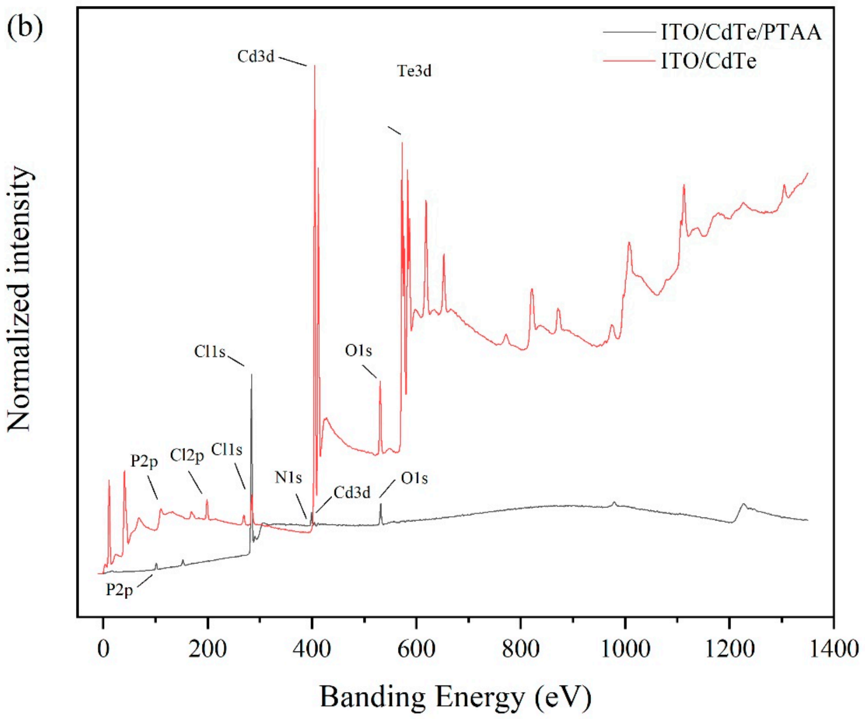

Figure S3. The XPS full spectrum of CdTe films and CdTe/PTAA films.
